# Unusual Presentation: Chronic Gastritis Caused by Sarcina ventriculi Identified via Helicobacter pylori Immunohistochemistry

**DOI:** 10.7759/cureus.76151

**Published:** 2024-12-21

**Authors:** Lingling Xian, Xuebao Zhang, Wei Xin

**Affiliations:** 1 Pathology, University of South Alabama College of Medicine, Mobile, USA

**Keywords:** chronic gastritis, cross-reactivity, helicobacter pylori, immunohistochemistry, sarcina ventriculi

## Abstract

This case report presents a rare occurrence of Sarcina ventriculi in a 15-year-old male patient with a clinical history of renal failure due to chronic rejection status post kidney transplantation, with persistent symptoms of diarrhea, nausea, vomiting, and fatigue. Despite exhibiting normal gastrointestinal mucosa upon endoscopy, biopsy analysis revealed chronic gastritis accompanied by the presence of Sarcina ventriculi in a tetrad arrangement across the stomach, duodenum, and distal esophagus. Interestingly, immunohistochemistry (IHC) staining targeting the Helicobacter pylori organism (H. pylori) also highlighted the presence of Sarcina ventriculi, suggesting a potential cross-reactivity between the two pathogens. Further investigation into the protein sequences and three-dimensional structures of H. pylori and Sarcina ventriculi revealed significant similarities, with a 65% protein sequence alignment and nearly identical configurations in the urease subunit. This finding suggests the possibility of cross-reactivity in diagnostic testing, particularly in IHC staining for H. pylori. This case highlights the importance of clinical awareness regarding Sarcina infections, particularly in symptomatic patients with seemingly normal endoscopic findings. The potential for cross-reactivity in IHC staining for H. pylori may offer valuable insights into the detection of Sarcina ventriculi, underscoring the significance of thorough diagnostic evaluation in such cases.

## Introduction

Sarcina ventriculi, also known as Clostridium ventriculi [1], is a gram-positive, anaerobic bacterium with unique tetrad morphology that thrives in acidic conditions. It was identified as a human pathogen 180 years ago [2] and has been spottily reported afterward [3]. It has been associated with delayed gastric emptying, emphysematous gastritis, and perforation [4-6].

Incidence of Sarcina ventriculi in patients with complex medical histories is extremely limited. Most reports are case studies or small case series, and no large-scale studies have systematically evaluated their prevalence. The lack of data is due to its rarity or underdiagnosis. Improved diagnostic tools, such as immunohistochemistry or advanced molecular techniques, might help clarify its true incidence in specific patient populations. In this report, we present a case of a Sarcina ventriculi infection identified in a 15-year-old male patient with chronic rejection and renal failure status post-kidney transplant, presenting with persistent diarrhea, nausea, and fatigue. Interestingly, we discovered that the H. pylori polyclonal antibody can clearly highlight Sarcina ventriculi through immunohistochemistry (IHC) when attempting to detect H. pylori in the stomach.

Furthermore, we investigated the structural and sequence similarities between H. pylori and Sarcina and identified a high degree of similarity in the urease subunits of the two bacteria. This study represents the first exploration of potential cross-reactivity and the underlying mechanisms between Sarcina ventriculi and H. pylori.

## Case presentation

**Figure 1:**
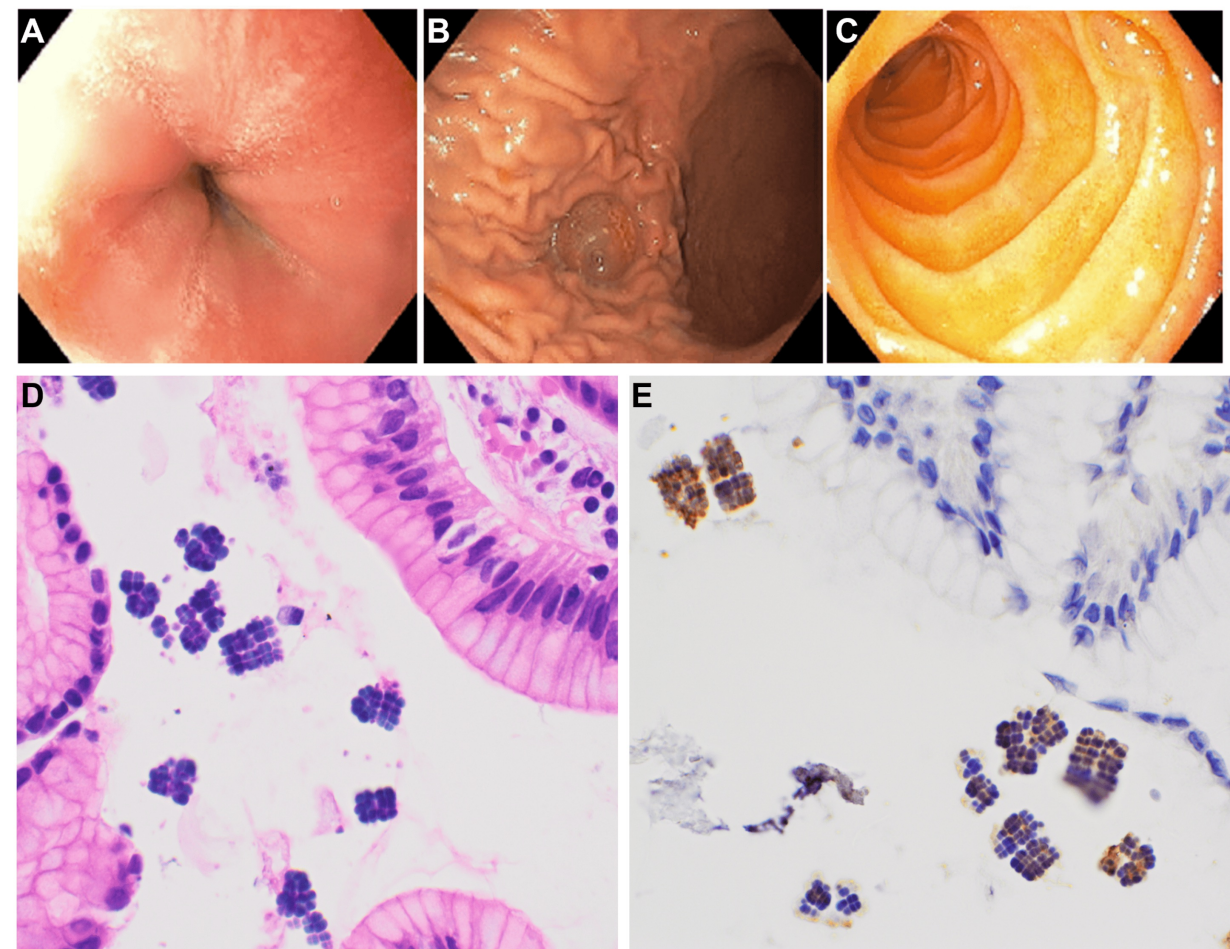
Esophagogastroduodenoscopy images of esophagus (A), stomach (B), and duodenum (C). (D) Gastric mucosa with Sarcina showing basophilic tetrad morphology (haematoxylin and eosin, x600). (E) Sarcina highlighted by H. pylori antibody by immunohistochemistry (IHC) staining (x600).

A 15-year-old male patient, who was undergoing hemodialysis due to chronic rejection and subsequent renal failure following a kidney transplant, presented with persistent symptoms of large volume watery diarrhea 8-9 times/day, nausea, vomiting, and fatigue for three months without knowing the etiology after extensive workups. Esophagogastroduodenoscopy, colonoscopy, and biopsy were performed. Despite normal findings in the gastrointestinal mucosa during endoscopy (Figure 1A-1C), microscopic analysis revealed mild chronic gastritis with no activity, the unique microorganisms arranged in tetrads were detected across the stomach (Figure 1D), duodenum, and distal esophagus, which morphologically consistent with Sarcina ventriculi. No H. pylori was identified by observing H&E slides. A subsequent IHC for H. pylori was performed. Interestingly, Sarcina ventriculi organisms were highlighted by H. pylori IHC staining by a rabbit polyclonal antibody (Cell Marque, Rocklin, CA, USA) and this antibody targets the general H. pylori bacterium, including the urease subunit (Figure 1E).

**Figure 2:**
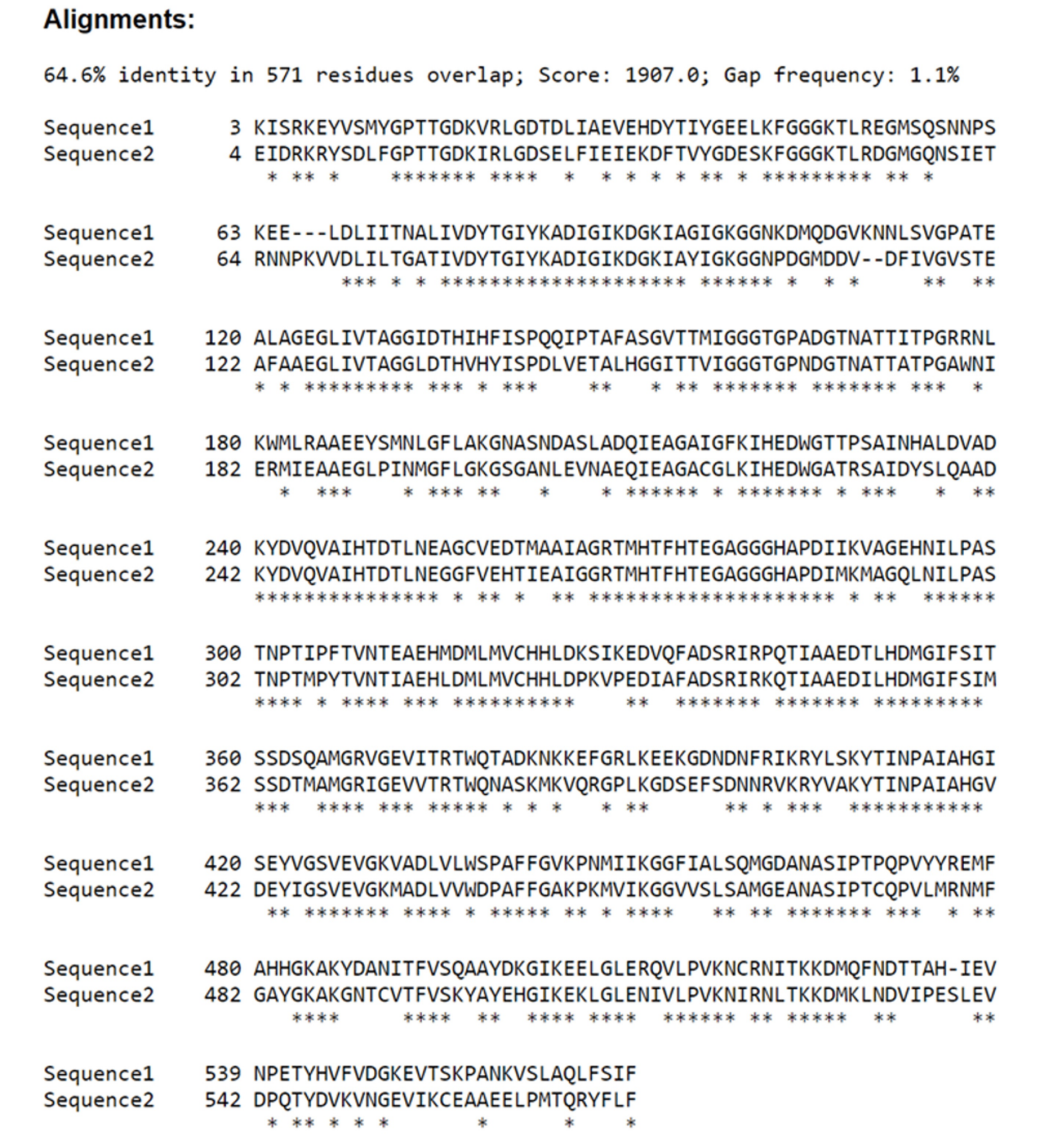
Protein sequence alignment of H. pylori urease subunit beta (sequence 1, 569AA), and Sarcina ventriculi urease subunit alpha (sequence 2, 572AA) through SIM-Alignment Tool for Protein Sequences.

**Figure 3:**
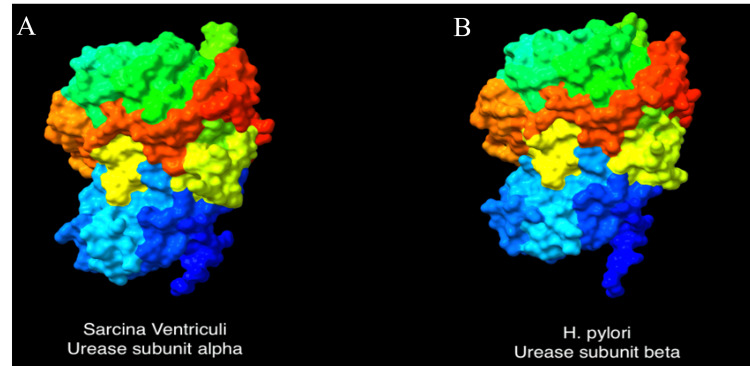
Highly similar 3D structure of Sarcina urease subunit alpha (A) and H. pylori urease subunit beta (B) from AlphaFold Protein Structure Database.

This finding prompted further investigation, which involved a comparative analysis of the protein sequences and 3D structures between H. pylori and Sarcina ventriculi through the online resources: UniProt (resource for protein sequence and annotation data) [7], SIM (Alignment Tool for Protein Sequences) [8] and AlphaFold Protein Structure Database [9]. We searched the sequence and 3D structure of H. pylori urease, flagellar proteins, cytotoxin-associated gene A (VacA), outer membrane proteins (OMPs) and neutrophil-activating protein (HP-NAP) of H. pylori, which are the most common antigens targeted by antibodies in H. pylori immunohistochemistry. Surprisingly, the comparison analysis revealed a substantial 65% amino acid sequence identity (Figure 2) and high similarities in the 3D configurations on urease beta-subunit of H. pylori and urease alpha-subunit of Sarcina ventriculi (Figure 3A, 3B). Other than urease, no overlap was identified between Sarcina ventriculi and H. pylori components, such as flagellar proteins, cytotoxin-associated gene A (VacA), outer membrane proteins (OMPs) and neutrophil-activating protein (HP-NAP), which are the common antigens targeted by antibodies in H. pylori immunohistochemistry.

Following antibiotic therapy with ciprofloxacin and metronidazole, the patient exhibited clinical improvement with reduced stool frequency at day 4, and ultimately achieved a clinical cure later. This successful outcome underscores the importance of accurate diagnosis and targeted treatment in managing gastrointestinal infections caused by uncommon pathogens like Sarcina ventriculi.

## Discussion

Sarcina ventriculi belongs to the Clostridiaceae family. Phylogenetic studies have classified Sarcina ventriculi within the genus Clostridium [10]. In 2016, there was a proposal to rename it Clostridium ventriculi [1], however, it ultimately retained its original name as the genus name Sarcina takes precedence over Clostridium.

Sarcina organisms can survive in very low pH environments where they form their distinctive morphological features. It was mainly isolated in the gastrointestinal tract (88%). Less common sites include the respiratory (5%), urine (4%), and bloodstream (3%) systems [11,12]. The significance of Sarcina ventriculi in pathology is a topic of debate, as it has been detected in patients who show no clinical symptoms, as well as in individuals with severe conditions such as emphysematous gastritis and gastric perforations. Symptomatic individuals have reportedly been effectively treated with antibiotics and proton pump inhibitors [13]. Sarcina has been described in both pediatric and adult patients. Pediatric cases, although less common, have a higher risk of being complicated by emphysematous gastritis or gastric perforation [6]. Common Sarcina-related symptoms are abdominal pain, distention, nausea or vomiting, diarrhea, and dyspepsia, but patients can also be asymptomatic. In this case, the young patient has a history of renal transplant, has undergone immunosuppressive treatment, recent chronic rejection, and chronic renal failure which can increase susceptibility to infections.

While both H. pylori and Sarcina ventriculi can be present in the stomach in a very low PH environment, there is no well-established association between the two bacteria in terms of causing specific diseases, protein sequence, and architecture or interacting with each other. Each bacterium is typically studied and understood in the context of the specific conditions which it is known to be associated with. In the presented case of a 15-year-old male patient with chronic rejection and renal failure post-kidney transplant, the identification of Sarcina ventriculi as the causative agent of persistent diarrhea, nausea, and fatigue highlights the importance of considering uncommon pathogens in patients with complex medical histories beyond the more typical pathogens such as H. pylori. The unexpected detection of Sarcina ventriculi using an H. pylori polyclonal antibody in immunohistochemistry (IHC) underscores the potential cross-reactivity and shared antigenic properties between these two bacterial species.

To investigate if there are structural and/or sequence similarities between H. pylori and Sarcina ventriculi, the online bioinformatics resources were used such as UniProt, the Alignment Tool for Protein Sequences (SIM), and the AlphaFold Protein Structure Database, and a comparative analysis of the protein sequences and 3D structures of H. pylori and Sarcina ventriculi was performed. Interestingly, a significant amino acid sequence identity (65%) and a high similarity in the 3D configurations of the urease subunit were identified. These results further support the biological and immunological similarities between these two bacterial species.

Urease is an enzyme that catalyzes the hydrolysis of urea into ammonia and carbon dioxide. This enzyme is produced by various bacteria, fungi, and plants. The most well-known example is Helicobacter pylori. Sarcina ventriculi is also a urease-producing bacteria. Urease plays a crucial role in the bacterium's ability to survive in the acidic environment of the stomach. By producing urease, H. pylori and Sarcina can convert urea into ammonia and carbon dioxide. The ammonia helps neutralize the acidic environment around the bacteria, allowing them to thrive and colonize the stomach lining. Urease is central to H. pylori metabolism and virulence. It is necessary for not only its colonization of the gastric mucosa, but also a potent immunogen that elicits a vigorous immune response.

It has been reported that certain monoclonal antibodies targeting H. pylori, particularly urease, may exhibit cross-reactivity with human gastric epithelial cells [14,15]. The autoimmune mechanism triggered by antibodies directed against H. pylori that interact with gastric epithelial cells has been proposed to play a role in the development of chronic gastritis associated with H. pylori [15]. The conserved urease subunit shared between H. pylori and Sarcina suggests that Sarcina may have similar autoimmune pathogenic potential in the gastrointestinal tract, which needs to be further investigated in the future. To our knowledge, this is the first reported investigation into the structural and sequence homology between Sarcina ventriculi and H. pylori, shedding light on a previously unexplored aspect of their pathogenesis.

Generally, histologic features of Sarcina are singular enough for a diagnosis on routine hematoxylin and eosin staining. Histologic diagnosis involves features like intense basophilic staining, tetrad, or octets arrangement [16]. However, a case series study from Johns Hopkins University Hospital showed that in most of their cases, the organisms were quite sparse and easily missed [17]. The Brown and Hopps stain is a differential stain that targets the polysaccharide layer of the bacterial cell wall, allowing for the selective visualization of Sarcina species. By utilizing this specific stain, pathologists can enhance the identification of Sarcina ventriculi and other Sarcina species in histological specimens, aiding in the accurate diagnosis of infections caused by these bacteria [17]. Additional methods, including polymerase chain reaction (PCR), sequencing of the 16S ribosomal RNA gene, pyruvate decarboxylase gene, or the IS-proTM technique, can confirm Sarcina ventriculi diagnosis [4, 17-20]. Currently, there is no specific antibody for Sarcina ventriculi, limiting targeted IHC detection. However, the observed cross-reactivity with H. pylori antibodies offers a promising alternative. Since H. pylori IHC is a widely available and routinely used technique in histology laboratories, leveraging this cross-reactivity could enhance the detection of Sarcina ventriculi, especially in cases where it might otherwise go undiagnosed. This highlights the potential for using existing, well-established diagnostic tools to improve recognition and reporting of this rare organism. Our findings in this case study open the possibility of utilizing H. pylori antibodies for screening both organisms within the same biopsy specimen, streamlining diagnostic efforts and enhancing clinical efficiency.

## Conclusions

This study emphasizes the importance of considering uncommon pathogens like Sarcina ventriculi in complex medical cases. The detection of Sarcina ventriculi using an H. pylori antibody reveals potential cross-reactivity and shared properties between these two bacteria, highlighting the need for accurate diagnosis and targeted therapy for gastrointestinal infections. Further exploration of structural and sequence similarities between these pathogens sheds new light on their pathogenesis, offering insights for future research and diagnostic approaches.
